# Managing Complex Anatomical Scenarios in Tavi: Evidence and an Institutional Perspective

**DOI:** 10.3390/jcm14217888

**Published:** 2025-11-06

**Authors:** Orlando Piro, Mattia Granato, Simona Covino, Emanuele Cigala, Mario Crisci, Riccardo Granata, Ida Monteforte, Paola Mocavero, Chiara Sordelli, Emilio Di Lorenzo

**Affiliations:** Division of Cardiology, Monaldi Hospital, 80131 Naples, Italy; orlando.piro@ospedalideicolli.it (O.P.); mattia.granato@live.com (M.G.); simona.covino@studenti.unicampania.it (S.C.); emanuele.cigala@ospedalideicolli.it (E.C.); mario.crisci@ospedalideicolli.it (M.C.); riccardo.granata@ospedalideicolli.it (R.G.); ida.monteforte@ospedalideicolli.it (I.M.); paola.mocavero@ospedalideicolli.it (P.M.); chiara.sordelli@ospedalideicolli.it (C.S.)

**Keywords:** transcatheter aortic valve implantation (TAVI), bicuspid aortic valve (BAV), hostile transfemoral access, aortic tortuosity and angulation, complex aortic anatomy

## Abstract

Transcatheter aortic valve implantation (TAVI) is the default therapy for most elderly patients with severe aortic stenosis, but outcomes in complex anatomy depend on imaging-guided planning and disciplined technique. This article aims to present our institutional approach, supported by the current literature, in managing several challenging anatomical scenarios. We focus on seven high-impact scenarios—bicuspid aortic valve (BAV), hostile transfemoral access, iliofemoral/aortic tortuosity, adverse aortic angulation, heavy annulus/Left Ventricular Outflow Tract (LVOT) calcification, small annulus, and risk of coronary obstruction—and propose a practical approach to minimize the risk of complications. In BAV, current generation transcatheter heart valves (THV) achieve favorable early outcomes when sizing accounts for supra-annular constraints and implantation depth is tailored. Transfemoral access remains dominant in contemporary registries, yet a meaningful minority of cases require adjunctive peripheral vascular intervention to enable THV delivery-system passage. In case of annulus or LVOT calcification, small annuli, complex aortic anatomy, high risk for coronary obstruction, and pre-procedural Computed Tomography (CT) allow for an accurate sizing of THV and tailored procedural planning. A structured, CT-driven pathway that links anatomic findings to specific facilitation and bailout steps can standardize decision-making and improve safety across these challenging scenarios. We strongly highlight the importance to build a network where most complex procedures are carried out in Valve Centers where expert operators are trained to manage high volume, high complexity, and difficult complications.

## 1. Introduction

Degenerative, non-rheumatic aortic stenosis (AS) has seen a significant increase in prevalence, becoming the most common valvular heart disease among the elderly in developed countries [1]. Prompt intervention is essential to reduce mortality, particularly in symptomatic patients or those with left ventricular systolic dysfunction secondary to valvular disease [2]. According to the 2025 ESC Guidelines for Valvular Heart Disease, transcatheter aortic valve implantation (TAVI) is the first-line treatment strategy for patients over 70 years of age. It may also be considered, following a comprehensive Heart Team evaluation, in patients at intermediate or high surgical risk [3]. The Heart Valve Network aims to reduce both underdiagnosis and undertreatment of AS by implementing specialized Heart Valve Clinics (for initial assessment and longitudinal follow-up) and advanced Heart Valve Centers. These centers should include both cardiology and cardiac surgery departments that offer 24/7 service availability, have expertise in multimodality imaging, and maintain high procedural volumes (approximately 100 TAVI procedures/year per hospital and 50/year per operator) in order to optimize clinical outcomes [4]. Furthermore, patients with anatomical or clinical features indicative of high procedural complexity should be referred to the most experienced Heart Valve Centers. High-risk anatomical features include challenging femoral access, severe aortic tortuosity, bicuspid aortic valves, extensive calcification, or low coronary ostia. Here, we present our center-based approach, supported by the literature, in managing the most challenging scenarios in patients referred for treatment by TAVI for severe aortic stenosis.

## 2. Methods

An electronic literature search was performed using PUBMED, MEDLINE, EMBASE, and SCOPUS to identify all studies evaluating the management, using TAVI, of patients with aortic stenosis and several complex anatomical scenarios. We focused our research by using the following main prompts: TAVI in stenotic BAV, BAV treatment, TAVI in stenotic BAV versus stenotic TAV, TAVI in ileofemoral hostilities, TAVI in hostile accesses, TAVI in angulated aorta, TAVI in aortic tortuosity, TAVI in annulus calcification, TAVI in LVOT calcification, TAVI in small annulus, and TAVI and coronary obstruction. We used no limits regarding study date. Based on the available evidence, we built an institutional, evidence-supported, center-based algorithm that we used in our daily practice to manage the different types of anatomical complexity in treating such patients using TAVI.

**BICUSPID AORTIC VALVE**

Bicuspid aortic valve (BAV) is the most common congenital heart disease, affecting 1–2% of the global population [5]. It is not only associated with valvular stenosis or regurgitation requiring intervention in both younger and older patients, but also with aortic dilation due to underlying genetic and hemodynamic abnormalities (e.g., turbulent blood flow and increased wall stress) [6].

Moreover, BAVs tend to be more calcified than tricuspid valves, likely due to local inflammatory response to endothelial damage [7].

There is lack of randomized studies comparing transcatheter and surgical valve replacement in BAV-related severe AS, as these individuals have been excluded from most randomized controlled trials (RCTs). A summary of the most important studies is displayed in Table 1. The Nordic Aortic Valve Intervention (NOTION-2) trial randomized 370 low-risk patients aged ≤75 years with severe symptomatic AS to undergo either TAVI or surgical aortic valve replacement (SAVR), including patients with both tricuspid and bicuspid anatomy. Although the composite rate of death, stroke, or rehospitalization at 1 year was similar between TAVI and SAVR, a higher rate of non-disabling stroke and paravalvular regurgitation (PVR) was observed in BAV patients undergoing TAVI [8].

Similarly, Elbadawi et al. compared in-hospital outcomes between TAVI and SAVR in 169 patients. They reported similar in-hospital mortality between the two groups, but TAVI was associated with lower risk of myocardial infarction, post-procedural bleedings, and vascular complications, as well as shorter length of stay. However, higher incidence of complete heart block and pacemaker implantation was noted in the TAVI subgroup [9]. Based on these findings, 2025 ESC Guidelines for Valvular Heart Disease recommend SAVR as the first-line treatment in this population, while TAVI may be considered in selected patients with increased surgical risk and suitable anatomical characteristics [3].

Despite this, in routine clinical practice, TAVI is often considered a good strategy in BAV patients. Several studies comparing outcomes after TAVI procedures in BAV versus tricuspid aortic valve (TAV) populations have yielded encouraging results (Table 1).

The PARTNER-3 Bicuspid Registry found no significant difference neither in the rate of the primary endpoint (a composite rate of death, stroke, and cardiovascular rehospitalization) nor its individual components between BAV and TAV patients treated with a balloon-expandible (BE) prosthesis [10]. Similarly, Makkar et al. compared outcomes for 3,168 propensity-matched pairs of BAV and TAV patients undergoing TAVI with BE protheses and found no difference in mortality and stroke rates at 1 year between the two groups [11].

The Low-Risk Bicuspid Study and the STS/ACC TVT Registry compared TAV and BAV patients undergoing transcatheter implantation of a self-expandible (SE) prosthesis. They found no significant differences in clinical outcomes (including all-cause mortality and stroke) and hemodynamic parameters (effective orifice area—EOA; mean gradient; maximum aortic velocity) at both 30 days and 1 year post-procedure [12,13].

We believe that TAVI can be a safe and effective therapeutic strategy in this clinical setting. However, a thorough pre-procedural stepwise approach is essential, starting with a detailed evaluation of the native valve anatomy and followed by a tailored selection of the bioprosthesis and implantation technique.

(1) *Anatomical BAV description*

Given the significant impact of anatomical characteristics on both the natural history of the disease and on the selection of the most appropriate treatment, an accurate morphological assessment of the valve is essential as the initial step.

**Figure 1:**
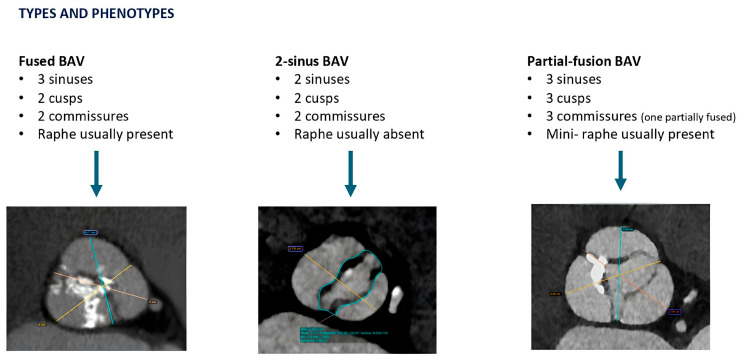
The three types and phenotypes of BAV are reported in the figure: Fused BAV, 2-sinus BAV, and Partial-fusion BAV. All the representative images come from our Institutional Database and refer to patients actually treated by TAVI in our unit.

The previously used Sievers classification [16] has recently been superseded by a more detailed system that takes into account the number of sinuses, cusps, and commissures and the presence or absence of a raphe. This classification can be further enhanced by evaluating the quantity and distribution of calcifications, along with a description of any associated aortopathy [17] (Figure 1). The most common phenotype is the fused BAV (90–95% of BAV), characterized by 3-sinuses, 2-cusps morphology, and with or without a raphe. The fusion of right and left cusps is the most common subtype (70–80%) and is frequently associated with dilation of ascending aorta (70% of BAV cases), likely due to increased wall stress on the outer curve. The degree of symmetry between fused and non-fused cusps depends on commissural orientation and significantly influences annular geometry: the more asymmetrical the cusps, the more elliptical the annulus tends to be. The BAVARD Multicenter Registry reported that BAV patients had larger annulus and ascending aorta diameters compared to TAV patients, but no significant differences were found in ellipticity index [18].

A comprehensive assessment of the aortic valve (AV) complex (from the annulus to the leaflet tips) is required. In addition to the classic tubular shape—where intercommissural distance (ICD) is equal to the annulus perimeter-derived diameter—a flared shape (ICD > annulus diameter) was identified in 52% of patients, while a tapered configuration (ICD < annulus diameter) was found in 13.8% of cases, with the narrowest point located approximately 4 mm above the annulus [18].

Finally, coronary anomalies are common in BAV patients, particularly anomalous origins or ectopic positions of the coronary ostia near the commissures. A thorough assessment of all these anatomical features is critical during preprocedural planning [19].

(2) *Choice of bioprosthesis and correct sizing*

Both Balloon Expandable (BE) and Self Expandable (SE) prostheses have been evaluated in BAV patients, demonstrating overall high procedural success rates (100% and 99%, respectively) [10,13]. However, bioprosthesis selection must be tailored to the anatomical characteristics of the native valve, carefully weighing the following advantages and limitations of each device:

- BE prostheses exert more radial force and have a lower stent frame height, which minimizes interference with coronary access and is associated with a reduced risk of permanent pacemaker implantation. However, accurate sizing is critical, particularly in valves with larger annuli or smaller annular structures, to avoid prosthesis malposition (pop-up or pop-down). Additionally, an increased risk of annular rupture has been reported, particularly in patients with significant annular or LVOT calcification, requiring greater caution in such cases [20].
- SE prostheses exert lower radial force, which may lead to non-circular or incomplete expansion of prosthesis in BAV anatomy. Nevertheless, this does not appear to affect leaflet function [21]. Conduction disturbances are more frequently observed after SE-valves implantation due to the compressive forces that push the high-framed stent of the prosthesis toward the cardiac conduction system (especially in R-L-fused valve phenotype with fibro-calcific raphes) [18]. SE valves have been associated with superior hemodynamic parameters in terms of lower thansprothesic gradients and larger effective orifice area, but at the cost of higher incidence of moderate-to-severe paravalvular leak (PVL) compared to BE valves [20,22]. Several sizing methods have been proposed to reduce procedural complications. One anatomical peculiarity of BAV anatomy is that the virtual basal ring (VBR) may not represent the narrowest point in the AV complex, suggesting that supra-annular structures should be taken into account in periprocedural planning and valve sizing. According to the BAVARD registry, ICD measured 4 mm above the annulus should be used for sizing in patients with tapered AV complex anatomy [18].

Tarantini et al. proposes to integrate supra-annular perimeter, raphe length, and the ante-raphe space to ICD measurement in order to obtain the measures of the so-called Virtual Raphe Ring (VRR). The comparison of VBR and VRR width points to a specific therapeutic strategy and prosthetic selection [23]. The “LIRA method”, proposed by Iannopollo et al., involves measurement of the so-called LIRA plane—defined as the plane cutting the major raphe (which is considered to be the anchoring point of the prosthesis) at its point of maximum protrusion in aortic root. The narrowest perimeter between the LIRA plane and traditional VBR is then used for sizing [24]. Another proposed method is the CASPER method, which incorporates calcium score, raphe length (less or greater than 50% of annular diameter), and calcium localization related to the raphe; however, it was validated on a small sample of patients [25].

Interestingly, the international BIVOLUTX registry found no statistically significant differences in clinical outcomes between the classic and combined sizing methods (incorporating both annular and supra annular structures).

In our view, the classic sizing method should be used in first instance, but a comprehensive structural analysis of the entire AV complex should always be performed and integrated into the final decision-making process. The choice between BE and SE valves should be guided by the extent and distribution of calcium, weighing the risk of major complications (particularly significant PVL and annular rupture).

(3) *Technical precautions*

Pre-dilatation is generally recommended in BAV patients to facilitate a better release and expansion of the prosthesis, especially in heavily calcified valves and prior to implantation of a SE valve. The size of valvuloplasty balloon should be based on the smaller diameter of the aortic annulus [26].

**Figure 2:**
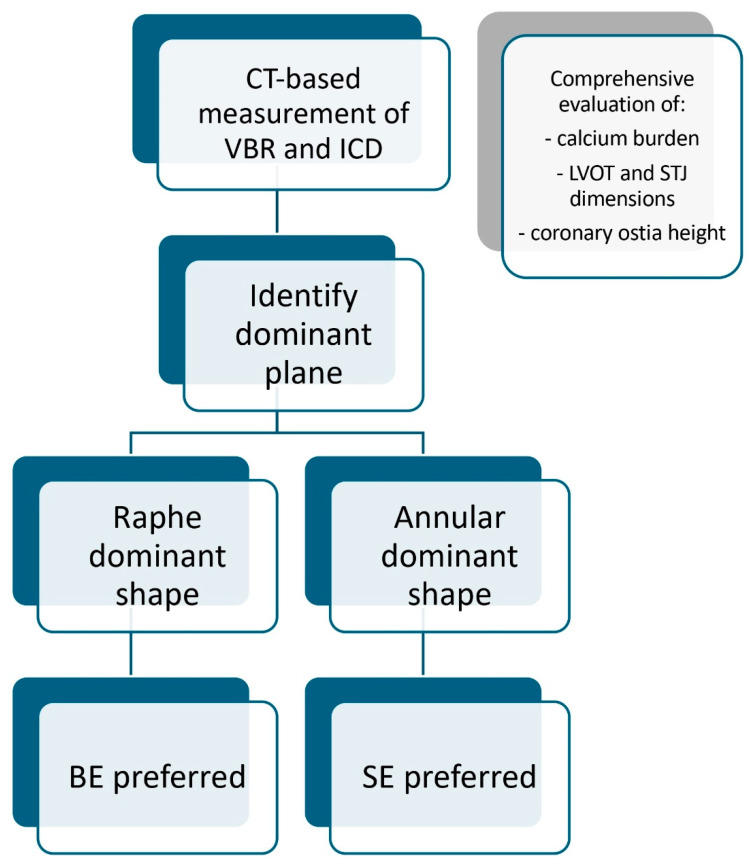
Our institutional step-wise algorithm is depicted. The choice of the preferred valve type strongly depends on the shape dominance: raphe vs. annular type. VBR: virtual basal ring; ICD: intercommissural distance; LVOT: left ventricular outflow tract; STJ: sino-tubular junction.

Valve positioning varies depending on the prosthesis used. BE-valves can be implanted using the standard “80:20” or “90:10” positioning strategy, just like tricuspid valves. In contrast, for SE-valves a higher implant position is preferred, using native valve’s cusp as anchoring point [27]. High-position implantation may enhance sealing by enabling the prosthesis’s double-layer skirt to expand more effectively above the annulus, thereby reducing paravalvular leak (PVL) and promoting optimal frame formation. Postdilatation is often necessary to improve prosthesis expansion, increase the EOA, and reduce PVL; sizing of the valvuloplasty balloon should be based on mean aortic annulus diameter derived from pre-procedural imaging. Our pre-procedural planning regarding patients with bicuspid valve anatomy suitable for TAVI is summarized in Figure 2**.** This is a simple stepwise approach that represents our institutional approach, which, although supported by the literature cited above, is not universally validated or guideline-based. The first step consists of measuring VBR and ICD at raphe level (raphe plane). If VBR is narrower than ICD (annulus-dominant shape), we consider the VBR as the landing zone of our prosthesis. In this case, our preference leans towards SE supra-annular valves, since their functioning should not be affected by VBR dimensions. If VBR is wider than ICD (raphe-dominant shape), we consider, as a landing zone, an anatomically upper plane at raphe level. Thus, we reduce the risk of sinus sequestration and coronary occlusion by opting for a BE valve (lower implant position).

## 3. Complex Vascular Access

In the first successful human TAVI, performed in 2002, Alain Cribier used an anterograde transseptal route via the common femoral vein, establishing procedural feasibility of extrathoracic peripheral access [28].

Subsequent developments brought multiple alternative accesses—transapical, transaortic, transaxillary, transcaval, and transcarotid—yet the retrograde delivery through the common femoral artery emerged as the preferred approach. Transfemoral (TF) access simplified catheter manipulation, enabled fully percutaneous treatment, shortened recovery, and steadily became the standard approach as operator experience and device profiles improved [29].

In contemporary practice, TF access was reported as the primary route in 99% of procedures in the European TAVI Pathway Registry; when TF access was not feasible, centers preferentially adopted extrathoracic alternatives (transaxillary/subclavian and transcarotid) [30]. In the United States STS/ACC TVT Registry (2019), TF access accounted for 95.3% of cases, while non-TF approaches comprised 4.7%, predominantly transaxillary/subclavian 2.5% and transcarotid 0.9%; transcaval was reported in 0.17%, with the remaining 1.1% representing intrathoracic approaches (transapical and transaortic), now uncommon [14].

In the Cleveland Clinic registry, up to 5% of TF-TAVI procedures required a peripheral vascular intervention (balloon angioplasty or stenting) [31] due to a challenging iliofemoral pathway; these cases were also associated with increased rates of vascular complications, which were standardized using Valve Academic Research Consortium 3 (VARC-3) definitions [32].

Pre-procedural CT is central to evaluate a possible hostile vascular access, which is defined by any of the following: (1) minimal luminal diameter < 5.0 mm in any iliofemoral or aorto-iliac segment; (2) minimal luminal diameter < 5.5 mm in the presence of either severe circumferential calcification (270–360°) or severe tortuosity (greatest curvature angle < 90° or a high tortuosity index); or (3) the coexistence of severe calcification and severe tortuosity anywhere along the route, irrespective of diameter [33].

Comprehensive whole-route CT analysis is needed, with a detailed report of minimum diameter segment-by-segment, focal stenoses, calcifications, kinking, aneurysm, dissections, and the relation between the access-site and the femoral head. Such a multiparametric CT assessment improves prediction of access-site complications beyond diameter alone [34].

Adequate caliber with non-circumferential calcium supports a standard fully percutaneous approach. After side-selection and definition of the puncture zone, each adverse CT finding should be used to plan specific preparatory steps [34,35].

(1) Approach for Hostile but Feasible Transfemoral Access

For TAVI to be successful in the setting of hostile vascular access, meticulous planning is mandatory. The common femoral artery should be punctured over the femoral head, ideally at mid-head, proximal to the bifurcation and away from anterior calcification, which may impair closure. Ultrasound-guided micropuncture improves first-pass success and reduces complications, and contralateral iliac angiography can clarify ambiguous fluoroscopic landmarks. In contemporary practice, percutaneous access is preferred; surgical cut-down is reserved for groins that are unsuitable for percutaneous closure [36].

In cases of severe focal iliac stenoses, a stepwise balloon predilatation is the best strategy to improves crossability, whereas heavily calcified, circumferential, or nodular calcification often necessitates the use of intravascular lithotripsy (IVL) to permit large-bore sheath passage and reliable closure. Peripheral IVL has been used to fracture intimal and medial calcium, and thereby allows for a smoother sheath passage, with high procedural success and acceptable safety, as observed in prospective and multicenter series [37,38,39].

For a long, diffuse, narrowing, and calcified iliac artery, an endoconduit strategy may be considered. Briefly, the placement of a covered stent-graft across the diseased segment, followed by high-pressure balloon dilatation (“pave-and-crack”), can create a controlled conduit for large-bore access, avoiding surgical intervention. Vascular series describe high technical success and durable mid-term patency with this approach when performed in experienced centers, showing feasibility in patients undergoing large-bore endovascular procedures, including TAVI [40]. The latter points to the importance to have an on-site Vascular Surgery Unit to face all the possible complex peripheral challenges.

For sheath management should be given priority to the smallest inner and outer diameters compatible for the selected transcatheter heart valve (VHT), systemic heparinization before upsizing, and advancement under angiographic roadmap with continuous hemodynamic and tactile feedback [41,42]. Persistent resistance after vessel preparation should prompt early reassessment to mitigate the risk of dissection or rupture [43].

**Figure 3:**
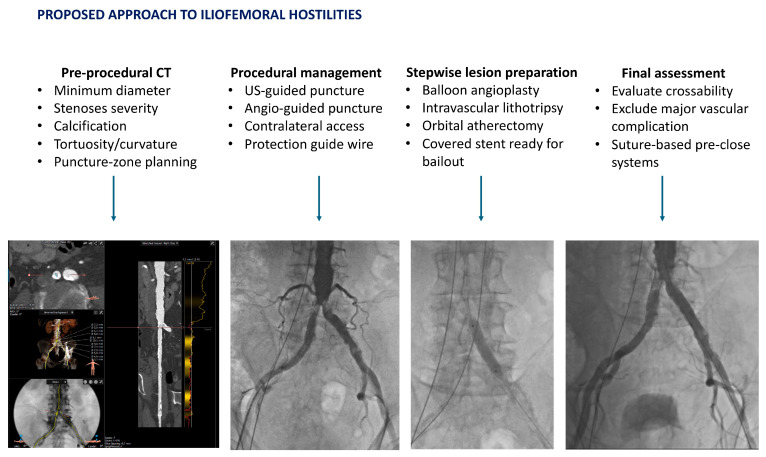
Our institutional approach for managing iliofemoral hostilities is depicted. Starting from a meticulous pre-procedural CT-evaluation, all the possible procedural steps, followed by a stepwise lesion preparation along with the final assessment, are proposed. All of the representative images come from our Institutional Database and refer to patients actually treated by TAVI in our Unit.

For hemostasis and arteriotomy closure, both suture-based pre-close systems and plug-based collagen devices are both acceptable options; comparative syntheses suggest broadly similar safety. Device selection should reflect vessel depth, calcification at the arteriotomy, operator preferences, and sheath size. Protamine reversal may be used to reduce access-site bleeding where appropriate [44]. Readiness for bailout is essential in hostile anatomy: suspected iliac or femoral rupture or flow-limiting dissection warrants immediate balloon tamponade followed by covered-stent implantation, with hemodynamic support as required [45]. A systematic approach, followed in our unit, is represented in Figure 3.

(2) When Transfemoral Is Prohibitive: Selecting Alternative Access

If pre-procedural CT and angiography indicate that transfemoral access is prohibitive and unsafe despite preparation, extrathoracic options should be chosen according to anatomy and center expertise. Transaxillary or subclavian access can be performed either percutaneously or via surgical exposure, according to operator experience. It provides reliable device control in suitable caliber vessels without circumferential calcium; registry analyses associate it with improved outcomes versus intrathoracic alternatives [46]. The transcarotid route offers a short, coaxial path, and has shown favorable outcomes in comparative cohorts when appropriate diameter and low plaque burden are confirmed [47]. Transcaval access provides a fully percutaneous venous-to-aortic solution with high technical success, and stroke and bleeding outcomes that are competitive with transaxillary in contemporary multicenter experience [48]. Intrathoracic routes, such as transapical and transaortic routes, are now rarely selected, given modern device profiles and improvement in alternatives, as previously described. Throughout, event definitions should follow VARC-3 to permit consistent reporting and comparison [32]. In Table 2, a simple step-wise approach is proposed.

### 3.1. Aortic Tortuosity

Tortuosity along the TF route is an anatomical determinant of device deliverability and access-site safety. CT-based studies identify tortuosity, together with calcific burden and vessel depth, as an independent predictor of vascular and bleeding complications after TF-TAVI, supporting multiparametric assessment beyond diameter alone [49,50].

Consensus on tortuosity quantification is present in the literature. Common CT metrics include length-based indices, such as the iliofemoral tortuosity score (ratio of centerline to straight-line length), and angle-based descriptors, such as the largest single bend and cumulative angulation; higher values correlate with increased access-site and bleeding events [50,51]. Case-based reports further illustrate successful TF-TAVI through severely tortuous, S-shaped, heavily calcified aorta when meticulous pre-procedural planning and support-wire strategies are applied [52].

When tortuosity and/or insufficient support limit advancement of the delivery, longer hydrophilic introducers, extra-stiff guidewires, and through-and-through rails, established via femoro-femoral access with snare externalization, can straighten the delivery path and stabilize advancement of sheath and valve [53,54,55].

Tortuosity is not confined to the iliofemoral axis. Abdominal aortic tortuosity associates with periprocedural complications in TF-TAVI, supporting evaluation of the entire thoraco-abdominal pathway in pre-procedural analysis [51]. In Table 3, CT metrics and procedural implications are summarized.

**HORIZONTAL AND ANGULATED AORTA**

Aortic angulation encompassing the ascending aorta and arch configuration is a determinant of deliverability and coaxial alignment. CT allows for quantitative assessment of the annulus–aorta angle along the centerline into the ascending aorta and arch, where operational thresholds > 50° commonly denote marked angulation. Early series linked greater angulation to reduced device success and increased technical difficulty, particularly with self-expanding valve, whereas newer-generation systems show attenuated effects on short-term outcomes [56,57,58].

Beyond a single global angle, landing-zone curvature and distal ascending/arch angulation influence implantation depth and repositioning requirements, especially with high stent-frame self-expanding systems [59]. The “gothic” arch is defined as an acutely angulated relatively short and transverse arch, and the main implications are consistent with tracking difficulty and reduced rail stability during TAVI [60]. The use of a long hydrophilic introducer can improve support, followed by a pre-shaped LV extra-stiff support. If the nosecone repeatedly tracks the outer curve, a contralateral femoral or radial snare-assisted rail can align and improve coaxial valve crossing [61]. When friction at the annulus or arch persists, balloon-assisted tracking (“buddy balloon” or “shoehorn” technique) can help the delivery system slide over the obstruction while maintaining wire stability [62]. If delivery remains unsafe despite these measures, operators should switch strategy rather than force advancement; an extrathoracic route that offers a shorter, more coaxial path, most commonly transaxillary/subclavian, is supported by contemporary multicenter data [46]. In Table 4, CT metrics and procedural implications are summarized.

### 3.2. Annulus and Lvot Calcification

CT characterization of the device-landing zone (DLZ) should describe calcium burden, distribution, and nodule morphology. Beyond global calcium volume, descriptors such as eccentric versus circumferential patterns, leaflet-root continuity of nodules, and protrusion into the LVOT are clinically informative; eccentric bulky nodules, particularly along the non-coronary cusp, have been linked to adverse device–tissue interaction [63,64].

Mechanistically, annular/LVOT calcium constrains radial expansion and impairs sealing. In balloon-expandable platforms, aggressive area-based oversizing in the presence of subannular/LVOT calcium increases the risk of annular or subannular injury; next-generation sealing skirts reduce—but do not eliminate—the impact of DLZ calcium on residual paravalvular leak (PVL). Regional calcium metrics outperform unweighted totals for predicting PVL [65].

Controlled, partial balloon inflation and careful post-dilatation with the minimum volume needed are reasonable to limit injury risk [66,67]. In case of residual moderate or severe PVL, a targeted and low-pressure post-dilatation is suggested [68].

Conduction disturbances after TAVI reflect interactions among implantation depth, membranous septum (MS) anatomy, and local tissue properties. Subannular LVOT calcium adjacent to the MS may amplify stress on the conduction system, but robust predictors across platforms are MS length and final implantation depth relative to the MS; a shorter MS and deeper implants consistently correlate with new conduction abnormalities and permanent pacemaker implantation (PPI) [69,70,71].

With self-expanding valves, a cusp–overlap implantation view is to be used in order to reduce pacemaker rates [72]. Baseline right bundle-branch block (RBBB) identifies a high-risk cohort that warrants a pacing and monitoring strategy beyond the default procedure [73]. In Table 5, CT descriptors, risks, and planning implications are summarized.

### 3.3. Small Annulus Anatomy

Small annulus, sinus of Valsalva, and STJ influence prosthesis selection, target implantation depth, and the risk for hemodynamic and structural complications [74].

Prosthesis–patient mismatch (PPM) remains a principal concern in very small annuli. Supra-annular self-expanding systems typically yield lower gradients and larger indexed effective orifice area (EOAi) than intra-annular balloon-expandable devices of similar nominal size; however, the clinical impact of moderate PPM appears attenuated after TAVI compared with SAVR, and severe PPM does not consistently translate into higher one-year mortality across contemporary series [75].

The combination of small annulus and subannular calcification increases the risk of annular/LVOT injury, particularly with aggressive area oversizing in balloon-expandable platforms; historical series identified eccentric calcific nodules and >20% area oversizing as key contributors.

A valuable approach is to prefer a conservative, CT-guided sizing, and to consider perimeter-based indices to reduce overexpansion risk [76]. When a balloon-expandable platform is selected, a slow, controlled inflation, and, if needed, a modest underfilling or a staged post-dilation (in order to achieve sealing while limiting rupture risk), are preferred [66]. In Table 6, CT findings and planning implications are summarized.

### 3.4. Risk of Coronary Obstruction

Coronary obstruction is uncommon, but catastrophic (≈0.6–0.7% overall), and carries ≈40% of 30-day mortality in affected patients; risk is higher in valve-in-valve and TAVR-in-TAVR procedures due to sinus sequestration [77,78,79].

A multiparametric CT approach outperforms single-parameter screening. The integration between coronary height and sinus of Valsalva diameters is crucial, and key metrics include the following: virtual valve-to-coronary distance (VTC), virtual valve-to-sinotubular junction distance (VTSTJ), and leaflet-to-STJ relationships. In native anatomy, VTC ≤ 4 mm consistently identifies a high-risk of coronary obstruction, while combinations such as VTC ≥ 3 mm with VTSTJ ≥ 3 mm, or (STJ height − leaflet length) ≥ +2 mm, characterize low-risk configurations [80].

The extended leaflet-to-ostium distance (ELOD) complements VTC by capturing effective clearance from the displaced leaflet edge to the coronary ostium; ELOD < 2 mm and leaflet length exceeding the mid-ostial height are associated with increased obstruction risk [81].

In valve-in-valve and TAVR-in-TAVR procedures, low STJ height with tall commissures predisposes to sinus sequestration. Predictive frameworks (e.g., the VIVID classification) and CT-based VTC/VTSTJ assessment identify candidates for leaflet-modification strategies or alternative treatment [82].

In the case of native-valve VTC ≤ 4 mm or ELOD < 2 mm, prevention and management include ostial protection in select cases with the “chimney” or “snorkel” stenting technique, advancing a workhorse 0.014-inch wire and positioning a non-compliant balloon or a deliverable stent at the ostium before THV deployment; in the case of coronary obstruction or sealing of the sinus, deploying a drug-eluting stent from the coronary into the aorta (in order to create a channel alongside the THV frame) can restore coronary flow. Multicenter registries shows high acute success rate, but a future re-access of the neo-sinus may be challenging [83]. Another preventive strategy is the Bioprosthetic or Native Aortic Scallop Intentional Laceration to prevent Iatrogenic Coronary Artery obstruction (BASILICA), consisting of leaflet laceration to maintain coronary flow in anticipated high-risk anatomies; contemporary series show promising effectiveness across native and bioprosthetic anatomies [84,85].

In Table 7, different correlation patterns between CT metrics and risk of coronary obstructions are summarized.

## 4. Conclusions

TAVI treatment in complex anatomical scenarios can be highly challenging. A meticulous stepwise approach is essential in order to minimize possible complications and to achieve the best and most durable results.

The lack of studies reaching any definitive conclusion and the numerous gaps in evidence prompted us to propose our institutional approach in managing several complex anatomies. Although not guideline-based, it is supported by the literature. Future studies are warranted to propose a universally validated approach in such challenging scenarios.

## Figures and Tables

**Table 1:** TAVI for the treatment of bicuspid aortic valve stenosis. Main randomized clinical trials, observational studies and registries are summarized. For each study, the design, type of device (when reported), numerosity, primary endpoints, and key results are highlighted.

Study	Design & Population	Device	Number	Primary Endpoints	Key Results
**NOTION-2 [8]**	RCT, low-risk ≤75 yrs with severe AS; includes BAV and TAV; TAVI vs. SAVR	Not specified	370	1-year composite: death, stroke, rehospitalization	Similar between TAVI and SAVR. In BAV with TAVI: higher non-disabling stroke and PVR
**Elbadawi et al. 2019 [9]**	In-hospital comparison of TAVI vs. SAVR in BAV	Not specified	169	In-hospital mortality, MI, bleeding, vascular complications, AV block, pacemaker	Mortality similar. TAVI had lower MI, bleeding, vascular complications, and shorter stay, but higher complete AV block and pacemaker
**PARTNER-3 [10]**	Registry, BAV vs. TAV treated with TAVI	Balloon-expandable (BE)	169	Death, stroke, rehospitalization	No difference between BAV and TAV for the primary endpoint or its components
**Makkar et al. 2021 [11]**	Propensity-matched analysis: 3,168 BAV–TAV pairs undergoing TAVI	Balloon-expandable (BE)	3168	1-year mortality and stroke	No difference between BAV and TAV
**Forrest et al., 2020 [12]**	Outcomes with self-expanding valves in BAV vs. TAV	Self-expandable (SE)	932	Procedural and clinical outcomes	Feasible with an acceptable safety profile in BAV
**The Low Risk Bicuspid Study [13]**	Observational, BAV vs. TAV undergoing TAVI	Self-expandable (SE)	145	Clinical outcomes and hemodynamic parameters at 30 days and 1 year	No differences in all-cause mortality, stroke, EOA, gradients, or Vmax; follow-up shows low rates of death or disabling stroke
**STS/ACC TVT Registry [14]**	National registry, BAV vs. TAV undergoing TAVI	Self-expandable (SE)	131	Clinical outcomes and hemodynamic parameters at 30 days and 1 year	No significant differences between BAV and TAV
**Yoon et al., 2020 [15]**	Impact of BAV morphology (raphe, calcification) on outcomes	Mixed	1034	Mortality and complications by phenotype	Calcified raphe and heavy leaflet calcification linked to higher complications and worse intermediate outcomes

**Table 2:** The main risk for each ileo-femoral hostile feature, along with the proposed solution to facilitate the “trans-femoral first” approach. When to convert and the preferred alternative approaches are also highlighted.

Hostile Feature (CT/US)	Primary Risk	TF-First Facilitation	When to Convert	Preferred Alternative
**Deep CFA with anterior calcium in site of puncture**	Access/closure failure; bleeding	US-guided micropuncture above bifurcation; choose suture vs. plug per depth/calcification	Inability to achieve stable pre-close or safe arteriotomy	Transcarotid or transaxillary/subclavian
**Isolated focal iliac stenosis** (non-circumferential)	Sheath impasse; dissection	Stepwise ballooning; consider short support sheath	Persistent impasse or recoil	Transcarotid or transaxillary/subclavian
**Circumferential iliac calcium** (270–360°) with borderline caliber	Rupture; dissection; failure to cross	Intravascular lithotripsy (IVL); long sheath; extra-stiff rail	Persistently non-crossable or high rupture risk	Transcarotid or transcaval (if anatomy favorable)
**Diffuse severe iliac stenosis with small caliber**	Rupture; inability to deliver	Endoconduit (“pave-and-crack”) with covered stent-graft, then dilatation; proceed TF	If endoconduit unsafe or fails	Transcarotid or transaxillary/subclavian; transcaval when venous/aortic anatomy favorable
**Marked tortuosity** (acute bends, long cumulative curvature)	Device kinking; vascular injury; failure to advance	Through-and-through rail (femoro-femoral or femoro-axillary), snare externalization; long sheath; gradual upsizing	Persistent inability to maintain rail or advance sheath safely	Transcarotid (short, straight path) or transaxillary/subclavian
**Access-site complication during case** (rupture/dissection)	Hemodynamic compromise; bleeding	Balloon tamponade, covered stent, reversal as appropriate; vascular surgery standby	Ongoing bleeding or limb-threatening flow	Definitive covered stent or surgical repair; switch access for completion
**Non-feasible TF after planning** (e.g., MLD < 5 mm with severe calcium/tortuosity)	Excess procedural risk	—	Up-front alternative access	Transcarotid or transaxillary/subclavian as first choices; transcaval in experienced centers

**Table 3:** The measurement of CT metrics of tortuosity, as outlined in each referenced published paper, associated with outcomes.

CT Metric	Measurement (CTA)	Representative Evidence	Association with Outcomes	Notes
**Iliofemoral tortuosity score (length-based)**	Centerline length ÷ straight-line distance	Mach 2021	Higher score → increased access-site and bleeding complications	Continuous risk; thresholds vary by study/device generation
**Largest single bend (angle-based)**	Minimum angle at tightest curvature	Mach 2021	More acute bends → tracking difficulty/adverse events	Complements length-based indices; interpret with calcium distribution
**Cumulative angulation**	Sum of sequential bend angles along the path	Perrin 2021	Greater cumulative curvature → vascular/bleeding events	Captures diffuse tortuosity
**Tortuosity index (global curvature)**	Study-specific centerline geometry index	Lux 2022	Higher index → more vascular/bleeding events	Combine with calcium scoring and vessel depth
**Abdominal aortic tortuosity**	Centerline curvature/angulation across abdominal aorta	Kinnel 2020	Predictive of complications in TF-TAVI cohorts	Evaluate entire thoraco-abdominal route

**Table 4:** Each CT-derived parameter of aortic angulation is associated with any possible procedural implication.

Parameter	CT Measurement	Operational Threshold	Procedural Implications
**Annulus–aorta angulation**	Centerline angle from annular plane into ascending/arch	≥50° for marked angulation	Greater technical difficulty; early signal for reduced device success; attenuated with newer systems
**Landing-zone curvature/distal angulation**	Local curvature and distal LZ angulation on CT	Study-specific (e.g., distal LZ~18°)	Influences implantation depth and repositioning (SE valves)
**Gothic arch morphology**	Qualitative classification on CT/MR	-	Acutely angulated short arch; unfavorable hemodynamics; tracking/rail challenges

**Table 5:** Selective plans are proposed for the optimal deployment of the device, according to different calcium localizations and distributions, in order to minimize complications.

CT Descriptor (DLZ)	What to Report	Primary Risk Association	Planning Implications
**Subannular/LVOT eccentric calcium** (often NCC side)	Cusp-wise location; nodule geometry; LVOT protrusion	Annular/LVOT injury with aggressive expansion (BE); sealing challenges	Avoid excessive oversizing; tailor platform and release strategy
**Regional DLZ calcium** burden and distribution	Cusp-specific volume/arc; annulus vs. leaflet continuity	Predicts PVR and need for post-dilatation	Plan sealing optimization; consider skirts and post-dilatation thresholds
**Protruding LVOT nodules** at/below annulus	Height beyond annular plane; interface with LVOT	Trigger for annular/subannular injury under balloon stress	Favor conservative expansion; reassess balloon volume/pressure
**Membranous septum**	MS length; implant depth relative to MS; adjacency of calcium	New LBBB/PPI risk after TAVI (platform-agnostic)	Target implant depth aligned to MS; consider cusp-overlap and recapture strategy

**Table 6:** Planning implications according to CT findings in small aortic annuli and roots.

CT Finding	Report/Quantify	Dominant Procedural Risk	Planning Implication
**Very small annular area/perimeter**	Indexed area; perimeter; eccentricity	PPM; residual gradients	Favor platforms with larger EOA (supra-annular) when appropriate; precise implant height
**Small root (reduced SOV/STJ)**	SOV diameter; STJ height/diameter; leaflet length vs. STJ	Coronary interaction and constrained expansion	Integrate coronary height, leaflet length, STJ geometry, and commissural alignment
**Small annulus with subannular/LVOT calcium**	Cusp-wise calcium; nodule geometry; LVOT protrusion	Annular/LVOT injury with aggressive expansion (BE)	Avoid excessive oversizing; weigh hemodynamic–mechanical trade-offs

**Table 7:** CT metrics and scenarios predicting coronary obstruction, along with the mechanisms and the implications, are summarized.

Metric/Scenario	How to Measure (CT)	Operational Signal/Threshold	Mechanism/Implication
**Coronary height and sinus dimensions**	Ostial height; SOV diameters; STJ height	Low LM (<~12 mm) and small SOV increase risk but are insufficient alone	Leaflet–ostium proximity; limited sinus washout
**VTC (virtual valve-to-coronary)**	Simulated THV frame; minimum distance to ostium	High risk when ≤4 mm; low risk when ≥3 mm with other favorable features	Clearance for displaced leaflet/frame
**VTSTJ and leaflet-to-STJ mismatch**	Minimum THV-to-STJ distance; leaflet length vs. STJ height	Low risk if VTSTJ ≥ 3 mm or STJ height − leaflet length ≥ +2 mm	Risk of leaflet pinning/sinus sequestration
**ELOD (leaflet-to-ostium)**	Distance from displaced leaflet edge to ostium	<2 mm associated with obstruction	Captures leaflet edge–ostium interaction beyond VTC
**ViV/TAVR-in-TAVR with low STJ**	CT with index THV; assess VTSTJ, sinus width, commissure height	High risk of sinus sequestration and global sinus occlusion	Coronary isolation despite adequate ostial height
**Preventive strategy: BASILICA**	CT eligibility (target leaflet[s], VTC/VTSTJ/ELOD)	Leaflet laceration to preserve coronary washout	Reduces anticipated obstruction in high-risk anatomies

## Data Availability

No new data were created or analyzed in this study.
